# Editorial: Tertiary lymphoid structures (TLS) in the tumor immune microenvironment

**DOI:** 10.3389/fimmu.2025.1555677

**Published:** 2025-01-23

**Authors:** Xinbo Gao, Xiangqin Zhao, Xuesong Li, Jin Zhang, Hui Zhao, Ying Ma

**Affiliations:** ^1^ Department of Pancreatic Cancer, Tianjin Medical University Cancer Institute and Hospital, National Clinical Research Center for Cancer, Tianjin’s Clinical Research Center for Cancer, Tianjin Key Laboratory of Digestive Cancer, Key Laboratory of Cancer Prevention and Therapy, Tianjin, China; ^2^ Department of Pediatric Oncology, Shandong Cancer Hospital and Institute, Shandong First Medical University and Shandong Academy of Medical Sciences, Jinan, Shandong, China; ^3^ Department of Orthopedics, Nanjing First Hospital, Nanjing Medical University, Nanjing, Jiangsu, China; ^4^ Department of Health Services Research, The University of Texas MD Anderson Cancer Center, Houston, TX, United States

**Keywords:** tertiary lymphoid structures (TLSs), tumor microenvironment (TME), immunotherapy, immune checkpoint inhibitors (ICI), solid tumors (ST), biomarkers, prognosis

Tertiary lymphoid structures (TLSs) (1, 2) are organized clusters of immune cells that develop within non-lymphoid tissues under specific conditions, including autoimmunity, chronic infections, and cancer. These structures resemble lymphoid follicles, typically featuring a core of B cells surrounded by T cells, along with dendritic cells, a supporting network of extracellular matrix, and specialized high endothelial venules facilitating lymphocyte entry. TLSs are thought to recruit and activate naive T and B cells within the tumor microenvironment (TME) via chemokine signaling, contributing significantly to the complex interplay of immune cells and tumor cells within the TME.

The TME in solid tumors comprises a complex ecosystem of tumor cells, stromal components, blood vessels, and immune cells. This environment plays a crucial role in tumor progression and its interaction with surrounding tissues. Tumor-infiltrating lymphocytes (TILs) exert a powerful influence within the TME, with cytotoxic TILs inhibiting tumor growth while certain suppressive or exhausted lymphocyte populations can promote it. TLS have been recognized as a significant source of TILs, and their presence often correlates with improved patient prognosis. However, our understanding of TLS function within the TME remains incomplete. Factors like TLS location, density, and maturity likely influence clinical outcomes, including survival and treatment response, across different cancer types. Furthermore, research into methods of manipulating TLS for therapeutic benefit is an area of active investigation, exploring their potential as immune niches to enhance existing and future cancer therapies. This Research Topic introduces a collection of articles in our Research Topic focused on TLS in solid tumors, exploring their anatomy, key features, immunological roles, and future research directions.

The fourteen articles in this Research Topic explore TLS across a range of solid tumors, including non-small cell lung cancer (Xu F. et al., Xin et al., Berthe et al., and Luo et al.), melanoma (Zhao et al.), gastrointestinal cancers (Yu et al.), colorectal cancer (Feng et al. and Xu Z. et al.), pancreatic adenocarcinoma (Gao et al.), cholangiocarcinoma (Shang et al.), and a meta-analysis across many types of solid tumors in 19 clinical trials (Jiang et al.). Several articles provide broader perspectives: You et al. and Ding et al. offer a comprehensive overview of TLS formation, maturation, localization, and heterogeneity, emphasizing the clinical implications of TLS heterogeneity in cancer patients. Zhao et al. elucidate the impact of immunogenic cell death-inducing chemotherapeutics on immune cell activation and TLS formation in melanoma.

These studies collectively highlight three key areas: the significance of TLS in predicting immunotherapy response and patient prognosis; the importance of assessing TLS maturity and density across different tissue types and spatial locations; and the crucial link between immune checkpoint pathways and TLS formation and maturation, with implications for understanding the mechanism of immune checkpoint inhibitors.

## 1 TLS in predicting immunotherapy response and patient prognosis

### 1.1 TLS and prognostic value in cholangiocarcinoma and pancreatic cancer

Cholangiocarcinoma (CCA), a malignancy of the biliary epithelium, carries a poor prognosis, hampered by the lack of reliable biomarkers for predicting treatment response and survival. Recognizing the role of tertiary lymphoid structures (TLS) as crucial microenvironments for anti-tumor immunity, Shang et al. investigated their prognostic value in a cohort of 471 CCA patients. Using H&E and immunohistochemical (IHC) staining to assess TLS maturity and composition, they observed varying degrees of TLS maturity and identified a four-gene signature (PAX5, TCL1A, TNFRSF13C, and CD79A) strongly expressed within TLS regions. High intratumoral TLS density correlated with improved overall survival (OS), while, interestingly, high peritumoral TLS density was associated with shorter OS. Similarly, a previous study (3) analyzed pancreatic cancer samples, identifying TLS-associated marker genes and developing a risk score model. This model stratified patients into high- and low-risk groups, with the low-risk group exhibiting increased immune cell infiltration and improved prognosis.

### 1.2 TLS in colorectal cancer: challenges and opportunities

While TLS are generally associated with favorable outcomes in several cancers, their role in colorectal cancer (CRC) is more nuanced. Although some studies have linked TLS presence to improved OS, progression-free survival (PFS), disease-free survival (DFS), and recurrence-free survival (RFS), Yu et al. highlight the lack of significant association between TLS and OS in CRC-specific subgroup analyses. This discrepancy may stem from the presence of pre-existing lymphoid tissues like GALT or Peyer’s patches, which could be misidentified as TLS. This approach could potentially enhance the prognostic utility of TLS in CRC. Furthering this line of inquiry, Xu Z. et al. developed a 14-gene TLS-related prognostic risk model, validated in TCGA and GEO datasets. They identified TLS-related subclusters and characterized hub genes, including PRRX1, a potential immunomodulatory factor and therapeutic target, whose expression was elevated in the TLS-positive CRC group. Their work showcases the combined power of bioinformatics, IHC, and multiplex immunofluorescence (MIF) for characterizing TLS and identifying clinically relevant markers.

### 1.3 TLS in non-small cell lung cancer: prognostic value and immunotherapy

Non-small cell lung cancer (NSCLC) remains a leading cause of cancer-related death, and while immunotherapy offers promising treatment avenues, robust prognostic markers are needed. Xin et al. revealed TLS in the current landscape of NSCLC and emerging immunotherapy strategies. Focusing on neoadjuvant chemoimmunotherapy, Xu F. et al. identified the platelet-to-lymphocyte ratio (PLR) as an independent predictor of TLS expression, with lower PLR correlating with higher TLS levels. Both systemic immune-inflammation index (SII) and TLS were independent prognostic factors, with high TLS and low SII associated with improved prognosis. Combining SII and TLS provided greater prognostic accuracy than either alone. Berthe et al. developed a multiplex IF panel to evaluate TLS maturity in NSCLC, finding that TLS relative area and CD21 positivity were strong prognostic indicators. Their TLS scoring system, incorporating TLS relative area, B cell density, and CD21+CD23- FDC density, demonstrated significant prognostic value.

### 1.4 TLS in pancreatic ductal adenocarcinoma and the complexity of TLS characterization

Pancreatic ductal adenocarcinoma (PDAC) is a highly aggressive subtype of pancreatic cancer, characterized by an immunosuppressive TME that contributes to immunotherapy resistance. While immune checkpoint inhibitors (ICIs) have shown limited efficacy in PDAC, other immunotherapeutic approaches are under development. Interestingly, the density of small nerve fibers within TLS aggregates has been linked to improved OS in PDAC. The complex architecture of TLS, comprising diverse immune and stromal cell populations, is essential for effective anti-tumor immune responses. However, the lack of a standardized TLS definition and the variety of assessment methods (H&E, IHC, MIF, gene expression profiling) contribute to variability in TLS classification and clinical interpretation. Characterizing TLS at a higher resolution, considering their functional, compositional, and spatial heterogeneity, is crucial for understanding their impact on patient survival.

## 2 TLS maturity and density across different tissue types and spatial locations

### 2.1 TLS formation, maturation, and characterization

TLS development is a multi-stage process involving fibroblast activation, immune cell recruitment, and maturation, as detailed by Gao et al. (4) Cytokines like IL-13, IL-17, and IL-22 play a role in the initial fibroblast priming by immune cells under inflammatory stress (5, 6). Histologically, TLS maturity was primarily distinguished by the presence or absence of germinal centers (GCs), crucial sites for B cell maturation and affinity maturation. Mature TLS, containing GCs, exhibit proliferating B cells, follicular dendritic cells (FDCs) expressing DC-LAMP, and markers like Ki67, AID, and BCL6. More recently, a three-tiered maturity model for lung cancer TLS has been proposed, classifying TLS as early (dense lymphocytic clusters without FDCs or GCs), intermediate (“primary follicle-like” with CD21+CD23- FDCs), and mature (“secondary follicle-like” with GCs) (7). This model underscores the importance of B cell maturation and humoral immunity in anti-tumor responses. However, TLS definitions vary across studies, with some relying on basic histological examination (H&E staining) and markers like PNAd or Ki67 (8, 9), while others employ more rigorous characterization based on distinct T and B cell zones, FDCs, and high endothelial venules (HEVs) (10, 11). This lack of standardization highlights the need for consistent criteria for defining and classifying TLS maturity.

### 2.2 Spatial heterogeneity of TLS in NSCLC

Xin et al. investigated the spatial distribution of TLS in non-small cell lung cancer (NSCLC), dividing tumor samples into intratumoral (IT), invasive margin (IM), and peritumoral (PT) regions. They further categorized TLS as early (E-TLS) or follicular (F-TLS). TLS density and the proportion of F-TLS were highest in the IT region, decreasing towards the IM and PT regions. Surprisingly, lower E-TLS density in the IM region correlated with better prognosis, possibly due to the suppressive immune environment at the tumor margin inhibiting TLS maturation. The IM region also showed increased infiltration of B cells, T cells, cytotoxic T cells, and macrophages, potentially explaining the correlation between these cell types and E-TLS density. E-TLS density in the IM region and TNM stage emerged as independent prognostic factors.

### 2.3 Contrasting TLS distribution and prognostic significance

In contrast to the findings in NSCLC, Feng et al.’s immunotherapy response scoring model in colorectal cancer revealed a different pattern of TLS distribution and maturation. A higher proportion of patients with higher scores, based on TLS characteristics, was observed in the peritumoral region compared to the intratumoral region. This scoring system, incorporating TLS distribution, quantity, and maturity, positively correlated with immunotherapy efficacy. This highlights the context-dependent nature of TLS and its prognostic significance. Furthermore, the role of TLS can vary across cancer types. While increased intratumoral TLS density is often associated with improved outcomes in intrahepatic cholangiocarcinoma, studies in other liver cancers have reported conflicting results. Both for hepatocellular carcinoma, Finkin et al. suggested that intratumoral TLS (12) could promote tumor progression, while Li et al. (25) linked peritumoral TLS to better prognosis. Similar discrepancies exist in breast cancer, bladder cancer, and gastric cancer, emphasizing the functional heterogeneity of lymphoid aggregates and the need for refined criteria to define functional TLS.

### 2.4 Spatial distribution and functional significance of TLS in different cancer types

The maturation state and density of TLS vary based on tumor type and spatial location, leading to diverse prognostic implications. This spatial heterogeneity is further exemplified in melanoma, where increased peritumoral mature TLS density is associated with improved survival (13). In PDAC, TLS are more frequently found at the invasive margin than in the tumor core (14). While one study showed a predominance of peritumoral TLS in PDAC samples (8), a more recent study highlighted the enhanced maturity, immune cell infiltration, and pro-inflammatory profile of the less abundant intratumoral TLS, associating them with improved survival (8). The dense, fibrotic stroma characteristic of PDAC may necessitate the close proximity of TLS to tumor cells for effective anti-tumor activity (15–17). These findings underscore the complex interplay between TLS location, maturation, and the tumor microenvironment in shaping clinical outcomes. Further research is needed to fully elucidate the factors influencing TLS function and their relationship with tumor progression in different cancer types.

## 3 Link between immune checkpoint pathways and TLS

### 3.1 The interplay between immune checkpoints and TLS formation

Immune checkpoint inhibitors (ICIs) targeting pathways like PD-1/PD-L1 and CTLA-4/CD80 have shown promise in cancer treatment. Studies have linked TLS abundance and spatial distribution to ICI response in cholangiocarcinoma (CCA). A previous study (3) stratified pancreatic cancer patients into high- and low-risk groups based on TLS marker gene expression. The low-risk group exhibited higher expression of both co-stimulatory immune checkpoints (e.g., CD28, TNFRSF4, CTLA4, CD40LG, ICOSLG, LAG3, PDCD1, TIGIT) and the inhibitory checkpoint CD276. This suggests that patients with abundant and well-distributed TLS might respond more favorably to ICIs.

### 3.2 TLS as a predictive biomarker and target for ICI therapy

Feng et al.’s work supports the link between TLS and ICI response. TLS presence could predict anti-PD-1 immunotherapy response in various cancers, including esophageal carcinoma, bladder cancer, melanoma, and head and neck squamous cell carcinoma (HNSCC), and may even be a direct target of PD-1 blockade (18–20). The association between high PD-1 expression at the invasive margin and TLS presence further suggests that context-specific PD-1 targeting within the tumor microenvironment may enhance efficacy (21). Xu Z. et al. also discussed a positive correlation between TLS and PD-L1 expression in colorectal cancer (CRC). These findings, along with evidence linking TLS to improved outcomes and immunotherapy efficacy in melanoma and breast cancer (22, 23), suggest that TLS can convert “cold” tumors to “hot” by enhancing immune recognition and clearance (24). Furthermore, recent research suggests that combining immunotherapy with strategies to promote TLS formation or maturation could amplify treatment efficacy.

### 3.3 Mechanisms of ICI influence on TLS

The abundance and maturity of TLS reflect a patient’s immune infiltration status, and ICIs have been shown to increase TLS abundance in several cancers. Ding et al. found that ICI response is linked to CXCL13-mediated recruitment of CXCR5+ B cells with high clonal diversity. Their *in vitro* data showed increased CXCL13 production in human peripheral blood mononuclear cells after anti-PD-1 treatment. This enhanced B cell infiltration and B cell receptor (BCR) diversity facilitates tumor antigen presentation, activating follicular helper CD4 T cells (Tfh) and tumor-reactive CD8 T cells. This influx of immune cells into the tumor microenvironment contributes to TLS abundance, maturation, and spatial organization. In essence, one mechanism of ICI action involves promoting antigen presentation by CXCR5+ B cells to activate CD4+ and CD8+ T cells.

### 3.4 The role of HEVs and immune checkpoint ligands

Luo et al. ‘s research on NSCLC revealed another ICI mechanism related to high endothelial venules (HEVs). Mature HEVs facilitate CD8+ T cell trafficking into the tumor, but immune checkpoint ligands (ICLs) expressed on these HEVs can hinder this process. Their ICL total score model demonstrated that HEV ICL expression predicts both CD8+ T cell infiltration and patient survival, with higher scores indicating poorer infiltration and prognosis. This suggests that ICIs can restore the function of specialized vasculature within TLS, enabling lymphocyte delivery into the tumor microenvironment and supporting TLS formation.

## 4 Future directions and limitations

Further research with large, prospective cohorts is needed to validate these findings and address limitations of previous retrospective studies, such as limited sample sizes and potential biases. Future studies should also incorporate more immunotherapy subgroups and address the challenge of comprehensively assessing TLS across the entire tumor. Larger sample sizes will help provide robust prognostic data and minimize the influence of individual differences and geographic variation. These efforts will advance the understanding of the complex relationship between TLS and ICI therapy, paving the way for more effective cancer immunotherapies.

## 5 Summary

**Figure 1:**
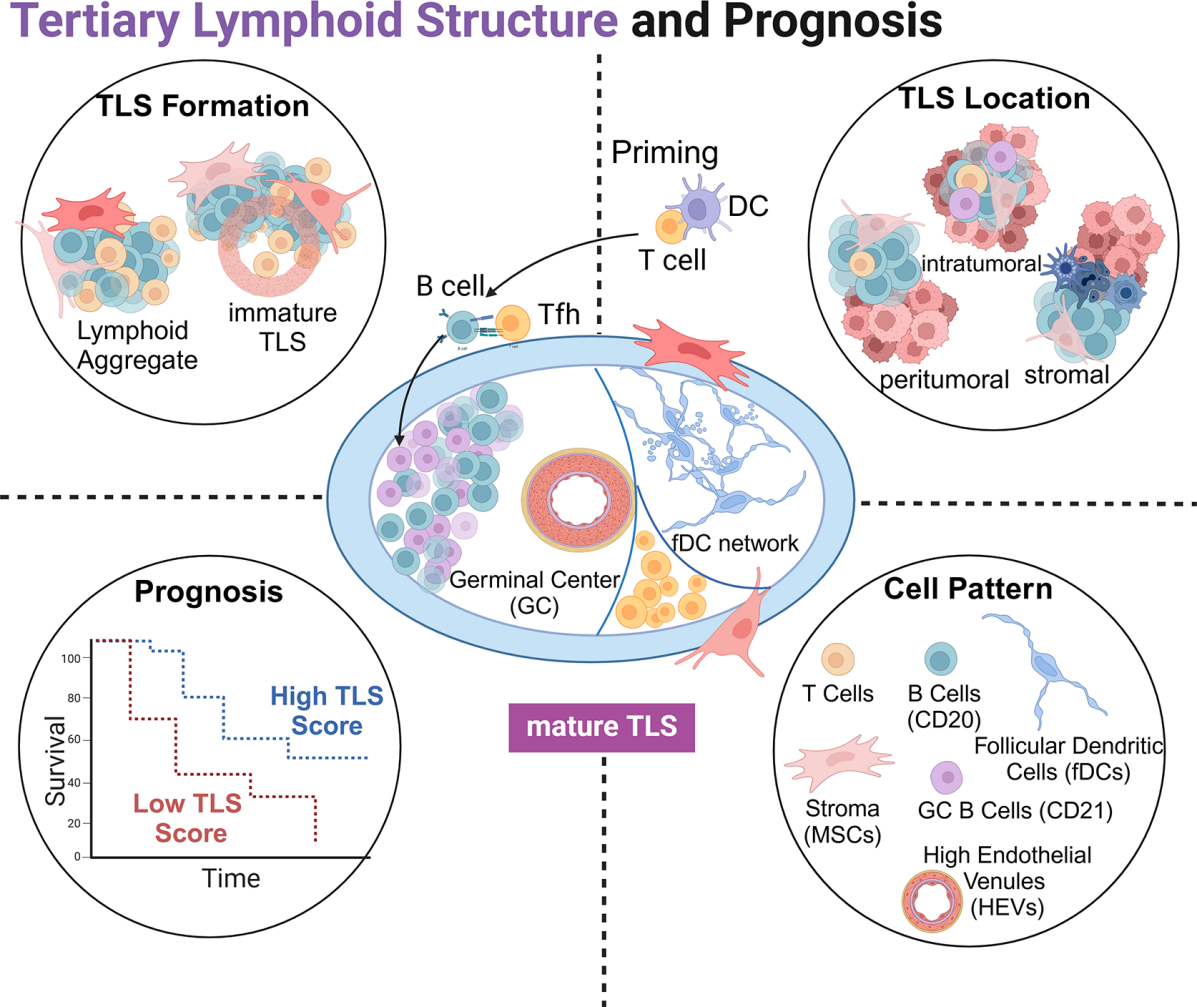
Schematic representation of tertiary lymphoid structures (TLSs): the structure of a mature TLS (center), the formation of TLS (top left), the relative positional relationship with tumor tissue (top right), TLS score with tumor prognosis (bottom left), and cell components involved in TLSs (bottom right).

The articles in this Research Topic provide a meaningful overview of the crucial relationship between TLS and ICI immunotherapy, highlighting the clinical significance of TLS in promoting anti-tumor immunity and predicting its prognostic value in solid tumors (**Figure 1**). Future mechanistic studies are needed to further explore this complex interplay.
